# *In vivo* antileishmanial activity of *Annona mucosa* extracts

**DOI:** 10.1590/0037-8682-0139-2019

**Published:** 2020-01-27

**Authors:** Janaína Paolucci Sales de Lima, Maria Lúcia Belém Pinheiro, Ivan Bezerra Allaman, Izaltina Silva-Jardim

**Affiliations:** 1 Universidade Federal do Amazonas, Departamento de Produção Animal e Vegetal, Manaus, AM, Brasil.; 2 Universidade Federal do Amazonas, Departamento de Química, Manaus, AM, Brasil.; 3 Universidade Estadual de Santa Cruz, Departamento de Ciências Exatas e Tecnológicas, Ilhéus, BA, Brasil.; 4 Universidade Estadual de Santa Cruz, Departamento de Ciências Biológicas, Ilhéus, BA, Brasil.

**Keywords:** Leishmaniasis, Leishmania amazonenses, Chemotherapy

## Abstract

**INTRODUCTION:**

Leishmaniasis, a disease caused by a parasite endemic to large areas of tropical and subtropical countries, is a growing public health problem.

**METHODS:**

Male BALB/c mice were infected with *Leishmania amazonensis* and treated with extracts isolated from *Annona mucosa*.

**RESULTS:**

Treated groups had significantly reduced footpad swelling. The group treated intraperitoneally with hexane extract showed footpad swelling similar to groups treated with Pentamidine® and Glucantime®. Groups treated with dichloromethane extract and hexane extract presented the recovering phenotype associated with reduced parasite levels.

**CONCLUSIONS:**

Extracts of *A. mucosa* are promising sources of novel antileishmanial compounds.

Leishmaniasis is a vector-borne disease affecting 400 million people worldwide. This disease is caused by different protozoan species in the genus *Leishmania.* Since 2013, the WHO Global Leishmaniasis program has reported the number of new autochthonous and imported cases to monitor trends in incidence. The Brazilian Ministry of Health has demonstrated the presence of *Leishmania amazonensis* in all regions of Brazil^1^ ^-^ ^2^.

Treatment of leishmaniasis with chemotherapy is unsatisfactory and has limitations. Leishmaniasis chemotherapy is currently based on daily intramuscular injections of pentavalent antimonials, diamines, and an antifungal polyene, all of which are toxic, expensive, generate resistance, and require long-term treatment^3^. 

In our previous work, *A. mucosa* extracts were assayed for antileishmanial activity against *L. amazonensis*, *L. braziliensis,* and *L. guyanensis* promastigotes. The dichloromethane extract of the leaves were promising; it inhibited the growth of *L. amazonensis* promastigotes and showed higher selectivity against parasites than the peritoneal macrophages^3^. In this study, hexane and dichloromethane extracts from *A. mucosa* leaves were evaluated in the treatment of experimental cutaneous leishmaniasis in BALB/c mice caused by *L. amazonensis*. This is the first report of topical formulations containing an *A. mucosa* extract.

*A. mucosa* Jacq (Annonaceae) leaves were collected from the Campus of the Federal University of Amazonas (UFAM) [coordinates: S 03°06’2.4”, W 59°58’27.7”], Manaus, Amazonas, Brazil in September 2007. A voucher specimen was deposited in the herbarium of UFAM under registration number 8148. Sample material was powdered after drying in an oven at 50°C for two days. The vegetal extraction procedure was performed as previously described^4^: dried and powdered (900 g) *A. mucosa* leaves were successively extracted with *n*-hexane and dichloromethane to yield hexane (18.4 g) and dichloromethane (42.4 g) extracts after removing each solvent. TLC indicated that the dichloromethane leaf extract contained the highest concentration of alkaloids. The dichloromethane extract (11.2 g) was then subjected to an acid-base extraction to produce dichloromethane alkaloid (0.25 g) and dichloromethane neutral (6.0 g) fractions. The alkaloid fraction was subjected to silica gel column chromatography eluted with hexane-dichloromethane (gradient from 100:0 to 10:90) followed by dichloromethane-methanol (gradient from 100:0 to 50:50), yielding 56 subfractions. Eluted subfractions were evaluated and pooled according to TLC analysis to create 6 fraction groups (GF1-GF6). GF3 (20.0 mg) was purified by preparative TLC eluted with hexane-acetone (60:40, three times) producing atherospermidine (2.0 mg) and liriodenine (10.0 mg)^3^, respectively.

The animal experiment was designed and performed in strict accordance with experimental protocols approved by the Animal Use Ethics Committee of the Research Institute for Tropical Pathology of Rondônia (Committee Number: 001/2011). Male BALB/c mice, 8-10 weeks of age (24-26 g), were kept in specific pathogen-free cages (n=8/cage) with free access to food and water and controlled temperature and light conditions. Animals were monitored each day during housing for health status. No adverse events were observed. The right hind footpad of each animal was injected with 10^6^ *L. amazonensis* promastigotes mL^-1^ (Strain: IFLA/BR/67/PH8) in 50 μL phosphate-buffered saline (PBS).

The clinical manifestation of *L. amazonensis* infection was monitored once a week by determining body weight variation and footpad thickness. Footpad thickness was measured using a caliper with an accuracy of 0.01 mm and expressed as the difference (in mm) between the infected footpad (iFP) and the noninfected footpad (niFP). Body weight variation describes the difference between the final weight (fw) and starting weight (sw) of individual mice in relation to the day of infection (Δ% = (fw - sw)/sw).

At five weeks post infection (p.i.), BALB/c mice were randomly allocated into eight groups for different treatment regimens. Group 1 was the positive control of infection, in which the infected group was not treated. Group 2 was the negative control of treatment, in which the infected group was administered topical treatment (t.t.) with Lanette Cream® (LC) as 5 µg/kg^-1^ body weight per day for 15 days. In group 3 (the positive control of treatment), the infected group was treated intraperitoneally (i.p.) with pentamidine isothionate on alternate days for 15 days. Pentamidine® was dissolved in 50 µL of PBS and administered to BALB/c as 4 mg/kg^-1^ body weight per day. Group 4 was the positive control of treatment, in which the infected group was treated i.p. with N-methyglucamine antimonate on alternate days for 15 days. Glucantime® was dissolved in 50 µL of PBS and administered as 100 mg/kg^-1^ body weight per day. In group 5, dichloromethane extract was incorporated in the formulation of 25 µg/g of Lanette Cream®. Infected group were treated by t.t. of dichloromethane extract cream (DEC) for 15 days as 5 µg/kg^-1^ body weight per day. In group 6, hexane extract was incorporated in the formulation of 12 µg/g of Lanette Cream®. The infected group was treated by t.t. of hexane extract cream (HEC) 5 µg/kg^-1^ body weight per day for 15 days. In group 7, the infected group was treated i.p. with dichloromethane extract (DE) dissolved in 50 µL of PBS and administered to BALB/c as 25 µg/kg^-1^ body weight per day on alternate days for 15 days. In group 8, the infected group was treated i.p. with hexane extract (HE) dissolved in 50 µL of PBS and administered as 12 µg/kg^-1^ body weight per day on alternate days for 15 days.

Close monitoring of body weight and footpad swelling continued until the end of experiment. Animals were euthanized two weeks after interruption of treatment, and single cell suspensions from the iFP were obtained. Parasite burden per footpad was determined by limiting dilution assays of infected footpads. It was not necessary to apply analgesics or anesthetics during the animal trials. The treatments had no apparent side effects.

The experimental design was completely randomized in split-plot time: 8 groups, 2 sampling times (7 and 14 weeks), and 8 replications for a total of 128 experimental units. Covariance analysis was conducted using the values obtained in the last week of pre-treatment as covariates for both the footpad swelling variable and the body weight variable. All assumptions were checked and when violated, the boxcox transformation was used. The Scott-Knott test was used when significant differences were detected between groups. The level of significance was set at 5%. R statistical software was used for analyses (R core team, 2019) with the aid of the stats package version 3.6 (R core team, 2019), nortest version 1.0-4, and ScottKnott version 1.2-7.

*In vivo* antileishmanial effects of *A. mucosa* extracts were investigated in an established *L. amazonensis* infection in BALB/c mice. Male BALB/c mice were housed in groups of four and given five days to acclimatize prior to infection. During housing, animals were monitored each day for health status. The variability of the animals during pre-treatment weeks is shown in Figure 1. Footpad lesions from BALB/c mice infected with *L. amazonensis* showed progressive lesions, beginning two weeks p.i. and reaching a mean of 2.54 mm four weeks p.i. (Figure 1A). Slight weight gain was observed in all animals, as expected based on animal age and housing conditions (Figure 2A).

**FIGURE 1:**
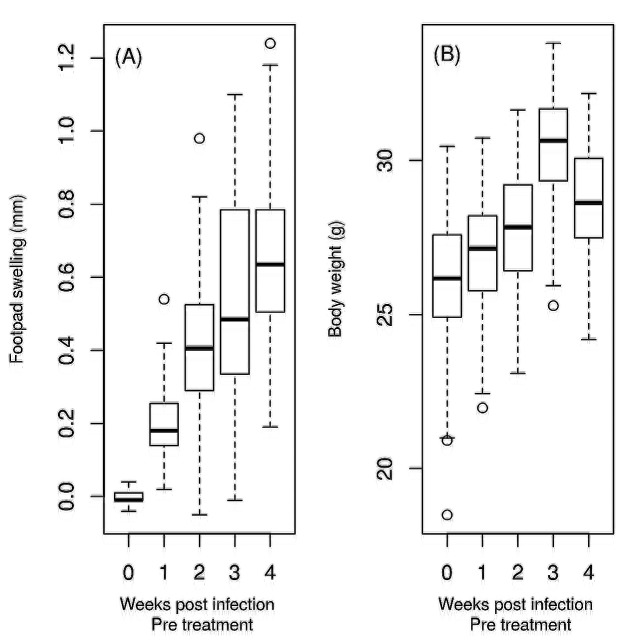
Variability of the animals as a function of time for the footpad swelling and body weight variables during the pre-treatment. The footpad swelling describes the difference between infected and non-infected footpad. The body weight variation describes the loss of weight in relation to the day of infection between the final weight and starting weight of individual mice. **(A):** Footpad swelling in pre-treatment. **(B):** Body weight in pre-treatment.

**FIGURE 2:**
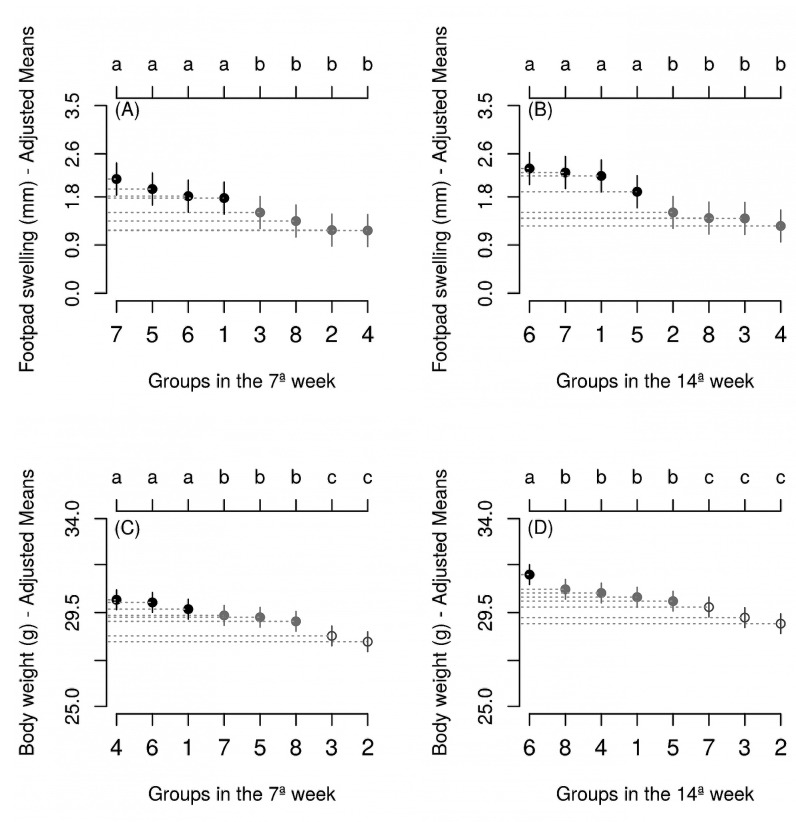
Footpad swelling and body weight variables of the different groups during the 7 **(A,C)** and 14^th^ **(B,D)** experimental weeks. Group 1 - Control, Group 2 - Lanette Cream®, Group 3 - Pentamidine®, Group 4 - Glucantime®, Group 5 - Dichloromethane extract cream, Group 6 - Hexane extract cream, Group 7 - Dichloromethane extract, and Group 8 - Hexane extract. Means with distinct letters differ significantly (Scott-Knott test, P <0.05). Vertical bars represent the 95% confidence interval. Averages were adjusted based on covariates of the last week prior to treatment.

Treatment regimens started five weeks after infection and lasted for fifteen days. T.t. and i.p. groups received applications daily and on alternate days, respectively. During treatment, animals were monitored daily for health status. There was a significant interaction between time and group (P <0.05) for both footpad swelling and body weight, and the groups were studied within each time.

In the seventh experimental week, the control group showed footpad swelling of 2.55 mm. Infected groups treated i.p. with Pentamidine and Glucantime for 15 days showed reduced footpad swelling by 45.51 and 36.85%, respectively. The Group Control and Groups DE, DEC, and HEC all showed similar footpad swelling. Group HE showed footpad swelling similar to the group treated with Groups Pentamidine®, Lanette Cream®, and Glucantime®, having a significantly lower mean (Figure 2A) that lasted until the end of the experiment (Figure 2B). Glucantime®, HEC, and Control groups showed similar body weights, with means higher than the other groups in the seventh week (Figure 2C). In the 14th week, group HEC was on average, superior to all other treatments, with groups DE, Pentamidine® and Lanette Cream® having significantly lower means (Figure 2D).

In the first post-experimental week, the control group showed footpad swelling of 2.80 mm (Figure 3A), which was an increase of 65.75% since the beginning of the experiment. The group treated i.p. with Pentamidine showed reduced footpad swelling by 18.57% compared to the control. There was a significant interaction between time and group for footpad swelling. Groups DE, HEC, and HE had similar means as the group treated with Pentamidine® (Figure 3A). Results were similar in the second post-experimental week (Figure 3B). Group HEC had a higher mean body weight than all other groups, independently of the first or second post-experimental week (Figure 3C).

Groups DEC and HEC had similar parasite loads as the control group and the Lanette Cream® group, with significantly higher means than the other groups (Figure 3D). The Pentamidine® group had the lowest mean parasite load.

There was a 4.74% and 13.55% reduction in parasite burdens in the infected footpads of animals with DE and HE, respectively, when compared to groups treated with Pentamidine® and Glucantime® (Figure 3D). Photographic documentation of three exemplary infected footpads per group demonstrated differences between the groups nine weeks post infection (Figure 3E). The recovering phenotype of the BALB/c mice treated with DE and HE correlate with reduced parasite levels. Reduced swelling after drug administration is frequently associated with therapeutic elimination of parasites in the footpad and is therefore used as the first clinical readout for drug efficacy^4^. However, the size of cutaneous lesions in the infected experimental animals is not necessarily an accurate reflection of the intensity of parasite burden. The size of these lesions is the result of a combination of the degree of replication and the resulting inflammatory response of the host.

**FIGURE 3:**
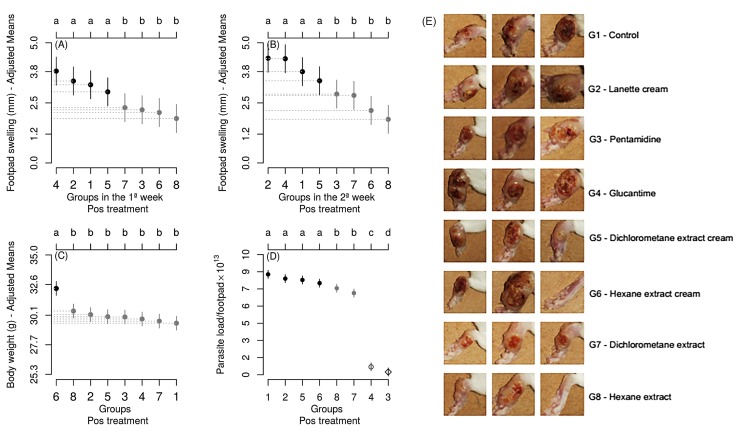
Footpad swelling **(A,B)** body weight **(C)**, and parasite load **(D)** post-treatment of the different groups. Group 1 - Control, Group 2 - Lanette Cream®, Group 3 - Pentamidine®, Group 4 - Glucantime®, Group 5 - Dichloromethane extract cream, Group 6 - Hexane extract cream, Group 7 - Dichloromethane extract and Group 8 - Hexane extract. Means with distinct letters differ significantly (Scott-Knott test, P <0.05). Vertical bars represent the 95% confidence interval. Averages were adjusted based on the covariates of the last week before the experiment (A,B,C). **(E)** Photographic documentation of the infected footpads of three exemplary mice per group, revealing the severity of disease progression in the treated and untreated groups nine weeks post-infection.

Recently, there have been significant improvements in available treatment options for leishmaniasis. Topical therapy is often indicated for CL when there are few lesions, and topical formulations offer the advantage of easy administration, fewer side effects, and cost-effectiveness in comparison to systemic treatment^5^ ^-^ ^6^.

Several studies have reported the efficacy of formulations for t.t. of CL against different species of *Leishmania in vitro* and *in vivo* models. However, the idea that systemic treatment is required to prevent the development of mucosal lesions has been questioned, and experimental results are encouraging. Few compounds of natural origin have been proven to be effective against CL infection in *in vivo* studies using topical treatment. Previous studies have shown significant decreases in parasite burden and lesion size in *L. amazonensis*-infected mice treated topically with dichloromethane extract of *Calophyllum brasiliense* Camb^7^, coumarin (−) mammea A/BB obtained from *C. brasiliense* ^8^, and podophyllin, obtained from *Indian podophyllum* or *Podophyllum peltatum* ^9^.

In the present study, HE resulted in a significant reduction in footpad swelling during and after treatment, whereas DE and HEC were observed only post treatment. Lipophilic compounds, such as the steroids sitosterol and stigmasterol, leaf wax palmitone, and the furolignans epi-membrin and epi-eudesmin, have been reported in hexane leaves extract of *A. mucosa* ^10^. The literature has also shown potent anti-inflammatory activity and weak anti-leishmanial action for sitosterol^11^, along with an *in vitro* anti-trypanosomatid activity for epi-eudesmin^12^. The effects of HE may be attributed to an anti-inflammatory response of the steroid sitosterol, which reduces the tissue damage caused by the immune system^13^, associated with its direct action and of other bioactive constituents on the parasite. Regardless, further *in vivo* investigations are required.

In contrast, the presence of oxaporphine alkaloids on leaf dichloromethane extract, mainly liriodenine, may partially justify the results for DE samples^3^ ^,^ ^14^. Although the mechanism of alkaloid action in *Leishmania* species is not yet understood, DNA topoisomerase inhibitors have been reported as promising anti-leishmanial drugs^15^, highlighting that liriodenine belongs to this group of substances^14^.

*A. mucosa* extracts were effective in treating leishmaniasis skin injury when applied topically and i.p. Results of this study are promising and stimulate the continued investigation, *in vivo,* of the isolated constituents of hexane and dichloromethane extracts of *A. mucosa leaves*, with the goal of potentially developing a leishmanicidal drug.
